# Pneumococcal Disease in High-Risk Adults in Lebanon: Expert Opinion

**DOI:** 10.3390/vaccines10101650

**Published:** 2022-10-01

**Authors:** Abdulrahman Bizri, Ahmad Ibrahim, Elissar Dagher, Madonna Matar, Malek Mohammed, Nizar Bitar, Paola Atallah, Rima Moghnieh, Umayya Musharrafieh, Zeina Aoun-Bacha

**Affiliations:** 1 American University of Beirut Medical Center, Riad El Solh P.O. Box 11-0236, Lebanon; 2 Al Makassed General Hospital, Tarik Jdide P.O. Box 6301, Lebanon; 3 School of Medicine and Medical Sciences, Centre Hospitalier Universitaire Notre Dame des Secours, Holy Spirit University of Kaslik, Jounieh P.O. Box 446, Lebanon; 4 Bahman Hospital, Haret Hreik P.O. Box 25-128, Lebanon; 5 Sahel General Hospital, Ghobeiry P.O. Box 99/25, Lebanon; 6 Saint George Hospital University Medical Center, Rmeil P.O. Box 166378, Lebanon; 7 Pulmonary and Critical Care Department, Hotel Dieu de France Hospital, University Medical Center, Saint Joseph University, Alfred Naccache P.O. Box 166830, Lebanon

**Keywords:** pneumococcal disease, high-risk adults, pneumococcal vaccine, adult vaccination, expert opinion

## Abstract

Pneumococcal disease affects people across all ages but is more prevalent in young children and the elderly. Despite the availability of the pneumococcal vaccine for adults, the disease burden and mortality associated with it remains a challenge. A few studies conducted in Lebanon have reported epidemiology of pneumococcal disease, concurring the high burden among adults and older adults in the region. The pneumococcal vaccine is a part of the routine immunization schedule for children, but there are no recommendations for adult vaccination. A medical advisory board was hence conducted in September 2020 to discuss the burden of pneumococcal disease (PD) among adults in Lebanon. The participants were experts from the fields of internal medicine, family medicine, hematology, cardiology, oncology, endocrinology, pulmonology, and infectious diseases. The experts reached a consensus that there is a need to take steps to increase the rate of adult vaccination uptake and create awareness among physicians, pharmacists, caregivers, and patients. The physicians should be trained on adult immunization and should actively discuss the importance of the pneumococcal vaccine, especially with high-risk adult patients. Implementing adult vaccination as a routine practice and involving various stakeholders to address the gaps can help in reducing the burden of pneumococcal disease in adults.

## 1. Introduction—Risk of Pneumococcal Disease in Adults

Pneumococcal disease is predominantly caused by *Streptococcus pneumoniae* and affects people across all ages [1,2]. The risk of pneumococcal disease increases distinctly with age [1,3]. Concurrent conditions such as alcoholism, cigarette smoking, chronic heart/liver/lung disease, diabetes, asthma, neuromuscular disorders, rheumatoid arthritis, Crohn’s disease, and any other condition or medicine that weakens the immune system, such as oral corticosteroids, increase the risk of contracting pneumococcal pneumonia or pneumococcal disease among adults. Patients with these conditions are classified as an “at-risk” population. The “high-risk” population includes patients with immunocompromising conditions such as chronic renal failure, nephrotic syndrome, congenital or acquired immunodeficiency (HIV), iatrogenic immunosuppression, malignancy, solid organ transplants, congenital or acquired asplenia, sickle cell disease, or other hemoglobinopathies. Patients taking immunosuppressive drugs and having a cochlear implant or a cerebrospinal fluid leak are also classified as “high-risk” [3,4,5]. Multiple risk factors impart a cumulative risk of contracting invasive pneumococcal disease (IPD) or community-acquired pneumonia (CAP) [4]. The increase in risk for pneumococcal diseases due to present comorbidities can be justified by the increased and dysregulated inflammation from aging, the delayed response of the immune system, and augmented expression of bacterial ligands in the lung [6]. A meta-analysis of 26 studies conducted in various countries reported the overall mortality rate due to IPD as 20.8%. Factors such as older age (>64 years old), septic shock, immunocompromising condition, underlying chronic diseases, solid organ tumors, alcohol abuse, nursing homes, and nosocomial infection were determined to be prognostic factors determining the mortality rate from IPD [7].

The risk of pneumococcal disease and the associated mortality remains a challenge despite the availability of the pneumococcal vaccine. The objective of this editorial article is to review the literature on the burden of pneumococcal disease in Lebanon among the adult population and the associated risk factors. The literature review has been supported by expert opinion from the authors. The authors have highlighted the gaps and unmet medical needs with respect to prevention of pneumococcal disease based on their clinical expertise.

## 2. Burden of Pneumococcal Disease in Middle East/Lebanon

In 2015, the Global Burden of Disease (GBD) study reported a total of 1,517,388 deaths due to pneumococcal pneumonia in all ages. The disease claimed 693,041 people aged ≥70 years [8]. A systematic review and meta-analysis conducted on articles published from Southern Europe reported an incidence of invasive pneumococcal disease (IPD) in adults to be 15.08 per 100,000 in Spain and 2.56 per 100,000 in Italy. The same study reported an incidence of 19.59 per 100,000 in Spain and 2.19 per 100,000 in Italy for pneumococcal pneumonia [9]. An Australian study reported hospitalization incidence due to pneumococcal pneumonia to be 274 per 100,000 population in 2011–2012 in adults aged ≥65 years. They also reported a case fatality rate of 6.1% between 2004 to 2012 [1]. A decline in the burden of pneumococcal disease has been reported by various studies due to the introduction of vaccination [2,9].

The overall mortality due to lower respiratory tract infections (LRTIs) has been reported to be 10% in the Middle East and North Africa (MENA) region, as opposed to 4% in developed regions of the world [10]. There are limited studies reporting the epidemiology and disease burden for pneumococcal disease in the region. However, the studies support a general consensus that there is a high burden of pneumococcal disease among adults and older adults in the region [11].

An epidemiological study conducted between 2006 to 2015 retrospectively reviewed the clinical course and outcomes of 103 adult patients infected with *Streptococcus pneumoniae* at Makassed General Hospital, Beirut, Lebanon. Among the 103 patients, 65% were ≥65 years of age and 35% had invasive isolates. The mortality rate was 21.6%; kidney disease and septic shock were significant mortality predictors. Superinfections, caused by extremely drug resistant (XDR) Gram-negative bacteria were developed by 19% of the patients and included ventilator-associated pneumonia (13%), hospital-acquired pneumonia (2.9%), bacteremia (1.0%), urinary tract infection (1.0%), and wound infection (1.0%) [12,13]. The serotypes identified in the 37 IPD isolates were 1, 3, 4, 7F, 9V/9A, and 19F, which are covered by the PCV13 and 23-valent pneumococcal polysaccharide vaccine (PPV23); 9N, 15B/C, and 33F is covered by PPV23; 16F, 18, and 29 are not covered by vaccines [13].

The Lebanese Inter-Hospital Pneumococcal Surveillance Program (LIPSP) conducted a prospective 6-year study between October 2005 to December 2011 at 78 hospitals distributed all over Lebanon. During the study duration, 257 isolates of *Streptococcus pneumoniae* were identified from patients who fulfilled the IPD criteria. There was a predominance of male patients (56%), mostly above 60 years of age (33.1%) followed by patients aged <2 years (24.1%) and patients between 21 to 60 years (16.3%). A total of 119 patients (46.5%) were diagnosed with pneumonia, 17.2% patients were diagnosed with meningitis and 14.8% had other diagnoses. The highest mortality was reported in patients above 60 years of age (25%) [14].

Further, the LIPSP highlighted the serotype distribution in Lebanon during the study period across all age groups. The serotypes identified were 19F (*n* = 31), 6 (*n* = 23), 14 (*n* = 18), 9V/9A (*n* = 13), 23F (*n* = 9), 4 (*n* = 9), 18C (*n* = 8), 1 (*n* = 16), 5 (*n* = 10), 7F (*n* = 6), 3 (*n* = 18), 19A (*n* = 15), 22F (*n* = 7), 33F (*n* = 6), 11A/D (*n* = 5), 9N (*n* = 5), 10A (*n* = 4), 12F (*n* = 4), 8 (*n* = 4), 15A (*n* = 3), 15 B/C (*n* = 3), 16F (*n* = 3), 23A (*n* = 2), 29 (*n* = 2), 29 (*n* = 2), 35B (*n* = 2), 38 (*n* = 2), and others (*n* = 12). The antimicrobial susceptibility testing reported 82.6% isolates to be susceptible to penicillin G using Clinical and Laboratory Standards Institute (CLSI) breakpoints. Among the isolates that were MDR, 19F (17 isolates) and 14 (5 isolates) were the most prevalent serotypes [14].

## 3. Immunization Recommendations and Challenges

In Lebanon, the 13-valent pneumococcal conjugate vaccine (PCV13) is included in the routine immunization schedule for children. The Lebanese Ministry of Public Health (MOPH) encourages vaccination for children with the approved vaccines. However, guidance for adult immunization is absent from the nation immunization schedule. Private practices and community pharmacies offer the pneumococcal vaccine to adults as per the international guidelines [13].

Until 2021, the United States Advisory Committee on Immunization Practices (ACIP) recommended the use of either one dose of PCV13 or one or two doses of PPSV23 in adults between 19 to 64 years who have not been previously vaccinated and carry an additional risk factor or have any underlying medical conditions. For adults aged above 65 years, PCV13 was recommended as one dose for those who have not been previously vaccinated and carry an additional risk factor or have any underlying medical conditions. The population above 65 years of age who do not have any risk factors or comorbid conditions can be vaccinated based on shared clinical decision-making. PPSV23 is recommended as a single dose for adults above 65 years of age who do not have a record of pneumococcal vaccination or do not have a documented medical history of past infection [15]. In its 2022 update, the ACIP recommends the use of either one dose of 15-valent pneumococcal conjugate vaccine (PCV15) followed by the pneumococcal polysaccharide vaccine (PPSV23), or one dose of the 20-valent pneumococcal conjugate vaccine (PCV20) in adults between 19 to 64 years who have not been previously vaccinated and carry an additional risk factor or have any underlying medical conditions. In adults aged above 65 years who have not been previously vaccinated, the ACIP recommends the use of either one dose of PCV15 followed by PPSV23 or one dose of PCV20 [16].

As of 2019, all 42 European countries have a vaccination program for adults, and 30 countries among these have recommended the PCV and/or pneumococcal polysaccharide vaccine (PPV) in the national vaccination policies for adults. The PCV is mandatory for specific groups in Czech Republic, Slovakia and Serbia, while the PPV is mandatory for specific groups in Serbia. The PCV is recommended for all adults in Austria, Belgium, Denmark, Finland, Greece, Hungary, Iceland, Italy, Luxemburg, Malta, Poland, Russia, Slovenia, and Spain. The PPV is recommended for all adults in Austria, Belgium, Cyprus, Denmark, Finland, France, Germany, Greece, Hungary, Iceland, Ireland, Italy, Luxemburg, Norway, Russia, Slovenia, Spain, Sweden, and the United Kingdom. Countries such as Bosnia and Herzegovina, France, Monaco, Montenegro, and North Macedonia have recommended the PPV while France and Lithuania have recommended the PCV for specific groups of patients. The other European countries have not recommended pneumococcal vaccination for adults [17].

There is limited updated information available on the disease burden of vaccine-preventable invasive bacterial diseases among adults from the Middle East and North Africa region. Although the available literature emphasizes the importance of enhanced surveillance efforts for these diseases within the region, there is an urgent need to develop and implement the surveillance systems to enable the integration of the healthcare sector with respect to epidemiology, laboratory, and data management [11]. This would support in developing robust vaccination strategies with more favorable outcomes. The other factors significantly influencing the clinical applicability of vaccination in adults include gaps in recommendations and limited or absent information on the risks and benefits of vaccination. The cost of vaccination and availability only in the private sector can lead to low voluntary uptake of vaccine by patients, especially those who are not covered by insurance.

## 4. Cost-Effectiveness of Pneumococcal Vaccines

Apart from the morbidity and mortality associated with vaccine-preventable diseases, they are also associated with economic burden. It was estimated that missed vaccination opportunities in adults above 50 years cost USD 26.5 billion for managing influenza, pneumococcal disease, herpes zoster, and pertussis in the United States [18]. A systematic review conducted on cost evaluation studies on pneumococcal vaccines for adults above 50 years noted that individually the included studies either concluded the pneumococcal vaccination (both PCV13 and PPSV23) to be either cost-saving or cost-effective as compared to no vaccination. The review evaluated 18 studies conducted in low- and middle-income countries [19].

There are no cost-effectiveness studies published from Lebanon. It is imperative to understand the economic burden of pneumococcal disease in adults, with and without comorbidities, to further convince policymakers and patients about the importance of vaccination.

## 5. Expert Opinion

A medical advisory board meeting was conducted in September 2020 with the objective to discuss the burden of pneumococcal disease (PD) among adults in Lebanon. The experts participating in the advisory board were set to identify the risk factors for PD in adults and also discuss the gaps and the unmet medical needs for PD prevention. The desired outcome of the meeting was to deliberate on the possible vaccination strategies that can be implemented in the Lebanese healthcare system to address the medical unmet need. A total of 10 experts participated in the discussion and belonged to the fields of internal medicine, family medicine, hematology, cardiology, oncology, endocrinology, pulmonology, and infectious diseases.

The first part of the discussion was regarding the characteristics of PD patients. Around 90.9% of experts agreed that *Streptococcus pneumoniae* is the most commonly associated pathogen with pneumonia, followed by *Hemophilus influenzae*. Patient-associated factors such as age (young children and adults >65), heart disease, diabetes, chronic lung disease, smoking, and cancer increase the risk of acquiring pneumonia. The majority of experts believed that chronic lung disease is the most common risk factor associated with pneumococcal pneumonia.

Around 10% of the patient population treated by the experts develop pneumonia. As per the experts, around 40% of the total community-acquired pneumonia (CAP) patients are hospitalized. Around 15% patients who are hospitalized catch hospital-acquired pneumonia, while around 7.5% of the hospitalized patients are admitted due to pneumococcal pneumonia. The median length of hospital stay was reported to be 7 days. All the experts agreed that the hospitalization duration or outcome varied based on the causative pathogen. Around 5% of patients are admitted to the intensive care unit (ICU) due to pneumococcal pneumonia and the mortality rate due to pneumonia was reported to be 4%. The experts reported that 27.5% viral pneumonia can be complicated by secondary bacterial infections.

All the experts agreed that pneumonia aggravates hyperglycemia, and hence results in poor glycemic control and even diabetic ketoacidosis in diabetic patients. Diabetes is also known to aggravate pneumonia and raises the risk of pneumonia-related hospitalization [20]. Chronic heart disease, especially congestive heart failure, also increases the risk of pneumococcal pneumonia in adults. Additionally, they are at an increased risk of hospitalization, requiring prolonged hospital stays, and carry a higher cardiovascular mortality risk [4]. In patients with stable angina or decompensated heart failure, pneumonia may trigger myocardial infarction. Patients who have a pre-existing respiratory disease such as chronic obstructive pulmonary disorder (COPD) and asthma may experience further respiratory complications due to pneumonia. Pneumonia also increases the risk of acute respiratory distress syndrome (ARDS) and ventilation requirement among patients. Oncology patients who are immunocompromised due to the nature of their malignancy or due to chemotherapy have increased risk of acquiring pneumonia (community- or hospital- acquired). This might hinder their cancer treatment and deteriorate performance status. Their pneumonia can grow out of control even with adequate antibiotic treatment. The other conditions which increase the chances of acquiring invasive pneumococcal disease (IPD) as per the experts include splenectomy, severe fulminant infection, and viral respiratory tract infections. Pneumonia is a major cause of death in patients with allogenic hematopoietic stem-cell transplantation (HSCT) [21]. Patients who contract pneumonia are liable to experience recurrent pneumonias in the future.

The summary of discussion questions on patient characteristics and their responses is presented in Table 1.

All the experts reported that they used chest X-rays for establishing a diagnosis of pneumonia at their center. Other diagnostic tools include complete blood count (CBC), culture, procalcitonin, multiplex PCR assay, C-reactive protein, urinary antigen, and computed tomography (CT) of the chest for pneumonia. Around 91% of experts agreed that culture and radiology should be performed before starting empiric antibiotic therapy. The experts reported that for hospitalized patients, diagnostic tools such as bronchoscopy and bronchoalveolar lavage can be used to determine the etiologic agent causing the pneumonia. There was a consensus that sputum culture, blood culture and urinary antigen detection for pneumococcus, which are generally used for patients with poor clinical outcomes, have good sensitivity. These tests expedite the process of identifying targeted antibiotic therapies which helps in the judicious use of antibiotics. Some experts highlighted the challenges of obtaining sputum culture in some cases, which limits the option in recognizing the causative agent and consequent targeted therapy. The good prognostic ability of the urine antigen test was stressed but the availability of this testing tool is not always guaranteed in laboratories across Lebanon. The summary of discussion questions on diagnosis and their responses is presented in Table 2.

All the experts recommended vaccination for adults and for patients against both viral and bacterial respiratory tract infections. The experts believed that the population above 65 years of age are the most affected with pneumonia, followed by children below 12 months (54.5%) and children aged between 12 months and 23 months. Overall, as per the experts, vaccination is accepted by patients, and the majority believe pneumococcal vaccination can be given based on age and risk factors. In clinical practice, the experts consider criteria such as age ≥ 65 years, COPD, asthma, lung cancer, bladder cancer, smoking, impaired splenic function, immunosuppression due to chronic use of immunosuppressive drugs, immunocompromising conditions, and cost, to recommend pneumococcal vaccination. The experts recommended training and education among health care professionals and the general population, besides establishing a national immunization program that aims to increase awareness about pneumococcal vaccination in adults. Media campaigns that can reach a high proportion of the population and utilization of social media platforms to communicate and share information about the vaccine can also help disseminate such messages. The summary of discussion questions on vaccination and their responses is presented in Table 3.

The burden inflicted by pneumococcal disease in adults can be improved by educating physicians, pharmacists, caregivers, and patients. There is a need to raise awareness about the critical role of vaccines in preventing and controlling communicable diseases in adults, especially in regard to pneumococcal and influenza infections. Strategies and efforts to enforce tobacco prevention and controls need to be emphasized at the individual level or as part of a comprehensive tobacco prevention program, along with other healthy lifestyle practices such as physical activity and regular exercising. The presence of underlying medical conditions, such as chronic lung and heart disease, liver and renal disease and immunocompromised conditions, are risk factors that place individuals at a higher risk of developing pneumococcal disease.

At the national level, health awareness campaigns and the use of social media platforms to send messages to encourage vaccination among the population are crucial in reducing the burden of disease. Such initiatives should be conducted in coordination with and under the guidance of the Lebanese Ministry of Public Health (MOPH). This should be carried out hand-in-hand with continuing medical education (CME) among physicians in order to stay updated on the most recent guidelines concerning prevention and management of pneumococcal diseases in adults. The experts agreed on the need to establish a national immunization program for adults in cooperation with and with the support of Lebanese medical societies. At the same time, adult immunization should be added to the national calendar so that MOPH can subsidize adult vaccines and insurance companies can reimburse the cost of vaccines. This may improve the rate of adult vaccination uptake in Lebanon. The MOPH has taken steps to vaccinate adults above 65 years of age, and who have comorbidities, for free upon presentation of medical reports. However, more efforts will be needed to vaccinate the at-risk adult population below 65 years of age.

Hospitals and medical centers can also contribute by enforcing preventive measures, implementing vaccination reminders via electronic medical records, developing guidelines on CAP management, ensuring standardized evidence-based treatments, and auditing performance of physicians. This will direct physicians on how and when to consider conducting additional analyses such as cultures, polymerase chain reaction (PCR), chest imaging, and serology to identify, manage and prevent CAP in adults in a timely manner. The regulation of antibiotic use and stewardship need to be implemented in all healthcare institutions and in the community for managing infections, including pneumonia. On a larger scale, developing a CAP registry and conducting a community based etiologic-epidemiological study can generate useful data and can add to the existing database about the prevalence, severity, etiology and burden of pneumonia in adults.

As per the experience of the experts, COVID-19 cases associated with viral pneumonia were greater in number than those associated with secondary bacterial pneumonia. Although the incidence of pneumonia associated with COVID-19 is less than that experienced with influenza or other viral infections, the association exists, and the patients are more accepting of the idea of receiving pneumococcal vaccination.

The experts believe that the management of pneumonia patients in Lebanon is challenging, especially for the elderly and patients with comorbidities. Patients with pneumonia who are referred to hospitals are often severely ill and present with extreme symptoms of breathlessness and respiratory failure. These patients might extend their illness and require more time to recover. The experts echoed that adequate choice of treatment would improve clinical courses and overall response. Patients can have a better chance of recovery and good outcome if they are diagnosed early and in a timely manner. The over-the-counter sale of antibiotics should be restricted to curb the spread of antimicrobial resistance. The lack of required diagnostic tools in some hospitals is an impediment to the management of these patients, and there is a need to fill this vital gap that impacts patient outcomes.

The experts also agreed that adult vaccination uptake rate is not satisfactory in Lebanon and is underutilized. They suggested ways to enhance the vaccination uptake rate in Lebanon, summarized in Table 4.

There is a need to generate real-world evidence in support of efficacy of the pneumococcal vaccination. The experts suggested vaccinating the high-risk population against viral and pneumococcal pneumonia and influenza during the ongoing COVID-19 pandemic. This can be further facilitated by a collaboration between pharmaceutical companies and MOPH to financially help vulnerable patients by bearing their vaccination costs.

There is an evident dearth of sufficient published studies from Lebanon enumerating the burden of pneumococcal disease in adult population. However, Hanna-Wakim et al. (2012) shed light on the epidemiology, serotypes, and antimicrobial susceptibilities of invasive *Streptococcus pneumoniae* isolate in Lebanon. Later in 2020, Moghnieh et al. published an epidemiology of non-invasive and invasive pneumococcal infections in Lebanese adult patients hospitalized between 2006 to 2015. However, adult vaccination is yet to become a routine practice. The coronavirus disease (COVID-19) pandemic caused by the SARS-CoV-2 virus boosted the conversation about the importance of vaccination across all age groups. As of September 2022, 43% (2.4 million people) of the Lebanese population has completed the COVID-19 vaccination as per initial protocol and 5.8% of the population (324,431 people) has been partially vaccinated [22].

The healthcare professionals should be trained on adult immunization, and it was deemed crucial to introduce preventive medicine in the medical curricula early on. At the clinical level, physicians should discuss and share information about vaccination in adults with their patients, especially with those who are at a higher risk of acquiring invasive diseases such as pneumonia. These gaps, if addressed, can impact the outlook and practices of adult vaccination in Lebanon, and thus help in reducing the burden of vaccine-preventable diseases such as pneumococcal diseases in adults, especially the high-risk population.

## Figures and Tables

**Table 1:** Expert opinion on patient characteristics affected by pneumonia.

Sr. No.	Questions	Responses from the Experts		
1	The most common pathogens associated with pneumonia	*Streptococcus pneumonia*	90.9%	
		*Haemophilus influenza*	36.4%	
		*Group A Streptococcus*	9.1%	
		*Staphylococcus aureus*	0	
		*Klebsiella pneumonia*	0	
		Other		
		- Viral infection	9.1%	
		- Influenza	9.1%	
		- COVID-19	9.1%	
		- Mycoplasma	9.1%	
2	Patients with high risk for acquiring pneumonia	Chronic lung disease	100%	
		Age (young children and >65)	90.9%	
		Oncology patients	90.9%	
		Heart disease	81.8%	
		Diabetes	81.8%	
		Smoking	81.8%	
		Other		
		- Autoimmune disease	9.1%	
		- Splenectomy	9.1%	
		- Multiple sclerosis	9.1%	
3	Patients with high risk for acquiring pneumococcal pneumonia specifically	Chronic lung disease	90.9%	
		Oncology patients	81.8%	
		Age (young children and >65)	72.7%	
		Diabetes	63.6%	
		Heart disease	54.5%	
		Smoking	36.4%	
		Other		
		- Splenectomy	18.2%	
		- Autoimmune disease	9.1%	
		- Multiple sclerosis	9.1%	
		- Alcohol abuse	9.1%	
		- Influenza infection	9.1%	
4	Percentage of your patients who developed pneumonia	Median (IQR)	10% (13%)	
		Minimum–Maximum	1–40%	
5	Percentage of CAP patients that are hospitalized	Median (IQR)	40% (35%)	
		Minimum–Maximum	9–70%	
6	Percentage of CAP patients that are treated as outpatients	Median (IQR)	50% (40%)	
		Minimum–Maximum	10–75%	
7	Percentage of hospitalized patients who acquire hospital-acquired pneumonia	Median (IQR)	15% (20%)	
		Minimum–Maximum	1–50%	
8	Percentage of patients who are hospitalized due to pneumococcal pneumonia	Median (IQR)	7.5% (25%)	
		Minimum–Maximum	2–50%	
9	The length of hospital stay of patients admitted with pneumonia (days)	Median (IQR)	7 (2)	
		Minimum–Maximum	4–15	
10	Is there a difference in the hospitalization duration/outcome as per causative organism?	Yes	11	100%
		No	0	0
11	Percentage of patients admitted to ICU for pneumococcal pneumonia	Median (IQR)	5% (11%)	
		Minimum–Maximum	2–20%	
12	The mortality rate due to pneumonia	Median (IQR)	4% (8%)	
		Minimum–Maximum	1–30%	
13	The percentage of viral pneumonia that can be complicated by secondary bacterial infections	Median (IQR)	27.5% (31%)	
		Minimum–Maximum	10–50%	

**Table 2:** Expert opinion on the diagnosis of pneumonia.

Sr. No.	Questions	Responses from the Experts	
1	What are the pneumonia diagnosis tools used in your center?	Chest X-RAY	100%
		CBC	90.9%
		Culture	90.9%
		CT chest	81.8%
		Multiplex/PCR	63.6%
		Procalcitonin	63.6%
		Other	
		CRP	18.2%
		Urinary Antigen for pneumococcus	9.1%
2	Are culture and radiology performed before antibiotic use?	Yes	90.9%
		No	9.1%
3	For hospitalized patients, are there any diagnostic tools used?	Yes	81.8%
		No	18.2%
		*If yes, specify:*	
		Bronchoscopy	66.7%
		CT chest	16.7%
		Bronchoalveolar Lavage	16.7%
4	What is the rate of positive microbiology in your pneumonia patient?	Median (IQR)	40% (33%)
		Minimum–Maximum	20–60%

**Table 3:** Criteria for vaccination: expert opinion.

Sr. No.	Questions	Responses from the Experts	
1	Do you usually recommend vaccination?	Yes	100%
		No	0
2	If yes, do you usually vaccinate your patients for viral or bacterial respiratory tract infections?	Viral infection only	0
		Bacterial infection only	0
		Both	100%
3	What are the age groups most affected with pneumonia?	<12 months	54.5%
		≥12–23 months	45.5%
		2–4 years	18.2%
		5 -9 years	9.1%
		10–17 years	0
		18–39 years	0
		40–49 years	0
		50–64 years	36.4%
		>65 years	100%
4	How do you judge your patients’ acceptance of vaccination?	Very unacceptable	0
		Unacceptable	0
		Neutral	0
		Acceptable	81.8%
		Very acceptable	18.2%
5	In your opinion, what is the scope of pneumococcal vaccination?	Age-based only	0
		Risk-based only	9.1%
		Both	90.9%
6	What type of awareness programs do you recommend for HCPs and for the public?	Training and education	81.8%
		Media campaign	63.6%
		National immunization program	81.8%
		Social media	63.6%

**Table 4:** Ways to enhance adult vaccination in Lebanon.

Unrestricted vaccine availability to pharmacists
Increasing awareness regarding the importance, health benefits and cost-effectiveness of pneumococcal vaccination
National adult vaccination program
Price reconsideration and providing the vaccine in dispensaries at a subsidized price
National immunization guidelines
Removing barriers to vaccinations, such as cost and availability in remote areas
Simplifying vaccination protocols in terms of the number of shots and time spacing between shots

## Data Availability

Not applicable.
